# Mitral degenerative valve leaflets suspected as primary valvular tumor: a case report

**DOI:** 10.1186/s12872-023-03131-z

**Published:** 2023-02-22

**Authors:** Shiqiang Wang, Huaidong Chen, Ximing Qian, Fan He

**Affiliations:** Department of Cardiac Surgery, Sir Run Run Shaw Hospital, School of Medicine, Zhejiang University, East Qingchun Road 3th, Hangzhou, Zhejiang Province China

**Keywords:** Valve degenerative disease, Primary valvular tumor, Echocardiography

## Abstract

**Background:**

Echocardiography plays an important role in the diagnosis of heart disease. Sometimes, however, it may also provide misdiagnosis information.

**Case presentation:**

We report a rare case of a misdiagnosis of primary mitral valvular tumor with severe regurgitation by preoperative echocardiography. During the surgery, the true lesion was found to be mitral valve leaflet prolapse due to degenerative mitral valve disease.

**Conclusion:**

For individual patient, the best clinical decision not only needs the extensive application of echocardiography, but also needs the combination of clinical symptoms and more auxiliary examination.

**Supplementary Information:**

The online version contains supplementary material available at 10.1186/s12872-023-03131-z.

## Background

Primary valvular tumor and valve degeneration are generally easy to distinguish. But in some special cases, they have a certain similarity in echocardiography. Especially for patients with atypical symptoms, it is often misdiagnosed one from another. Here, we report a case of mitral valve degenerative disease suspected to be diagnosed as primary valvular tumor.

## Case Report

**Fig. 1:**
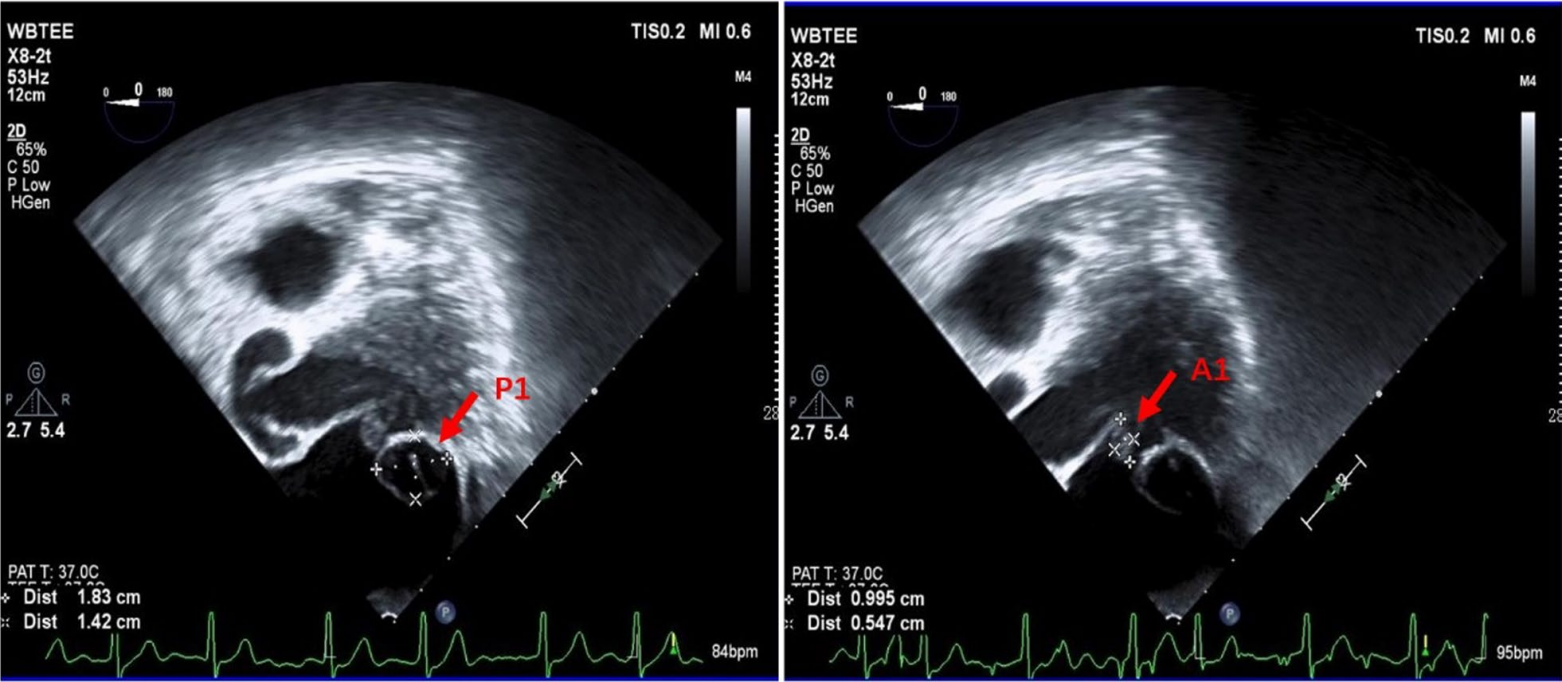
Transesophageal echocardiography showed that multiple cystic structures with visible separation were on the mitral valve leaflets. The larger one was located in the P1 area, about 18.3 × 14.2 mm in size, and smaller in the A1 area about 9.9 × 5.4 mm

**Fig. 2:**
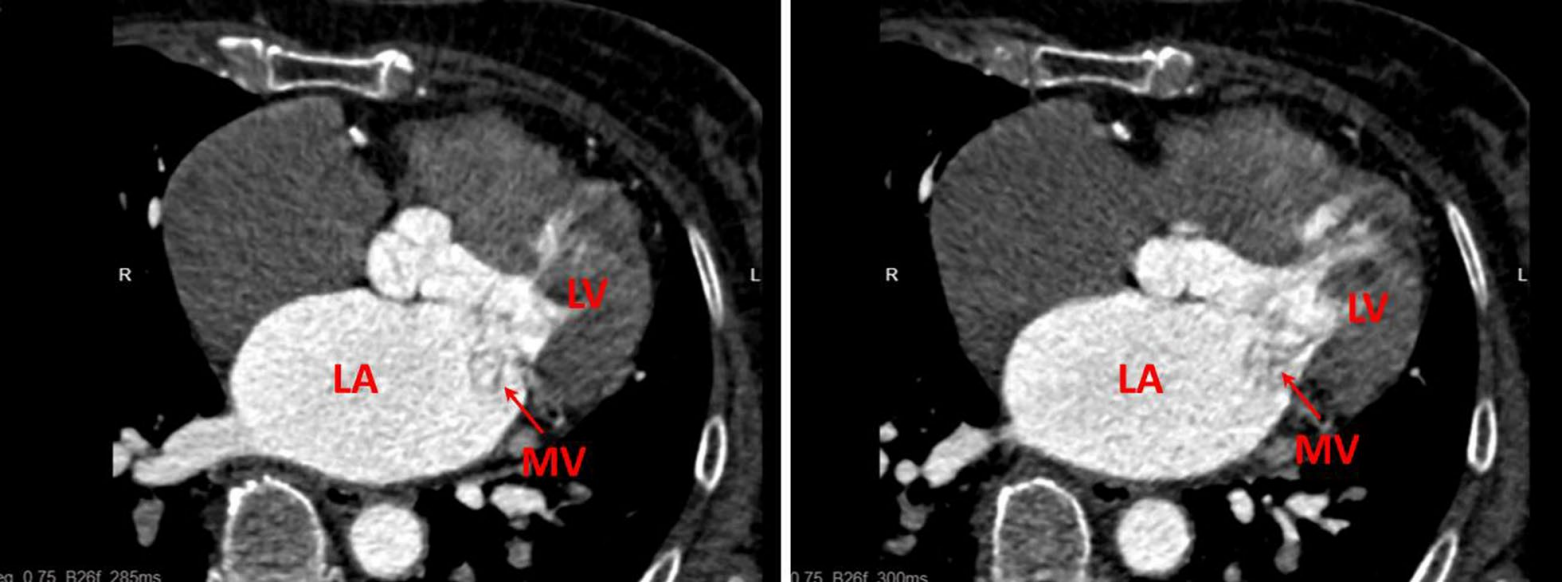
Coronary computed tomography angiography showed polycystic structures at the position of mitral valve

**Fig. 3:**
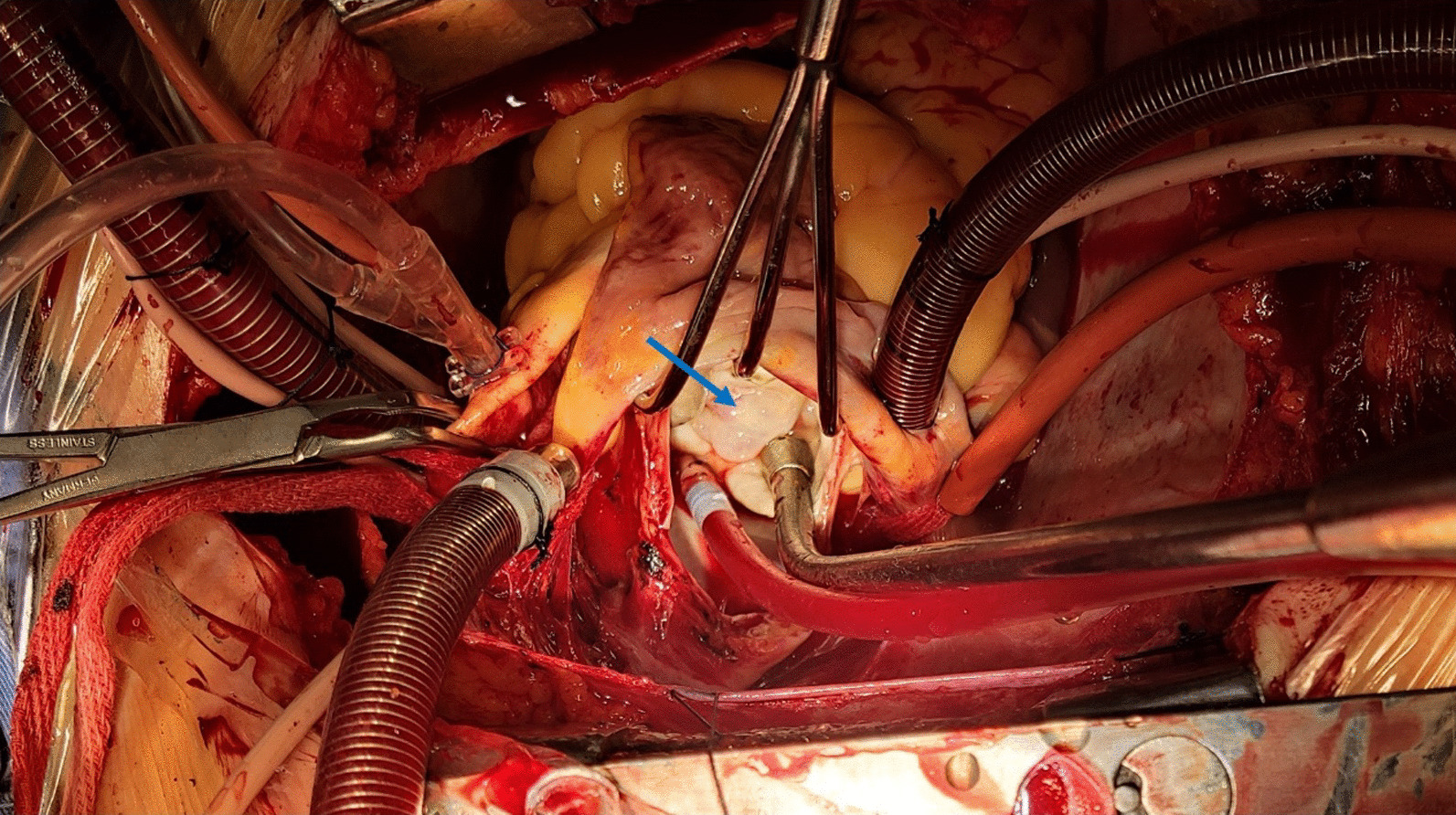
Mitral valve pathological changes during operation. The blue arrow indicates the mitral valve prolapse leaflet tissue

A 68-year-old female patient presented to the emergency department of our hospital with recurrent chest tightness and shortness of breath. On admission physical examination, systolic murmurs could be heard in the apex of the heart. The patient had no other comorbidities except hypertension. Transthoracic echocardiography revealed severe mitral regurgitation and mitral leaflet cystic nodules, suspected to be benign tumor or infective endocarditis with vegetations. Transesophageal echocardiography revealed multiple cystic structures with significant separation in the A1, C1, and P1 leaflets by the mitral Carpentier nomenclature [1]. The cavity was filled with blood and connected with the left ventricle. The volume of the cystic structure was enlarged in the systolic phase and decreased in the diastolic phase of the heart. The larger one was located in the P1 area, about 18.3 × 14.2 mm in size, and smaller in the A1 area about 9.9 × 5.4 mm (Fig. 1 and Additional file 1: videos 1–3). The conclusion of echocardiography showed that mitral valvular leaflet tumor should be considered first after excluding infective endocarditis. The patient experienced some examinations related to infective endocarditis, such as cranial Computed tomography (CT), brain magnetic resonance imaging (MRI) and blood culture, etc. But the results did not fulfill the modified Duke criteria for the clinical diagnosis of infective endocarditis. Preoperative coronary Computed tomography angiography (CTA) showed no obvious abnormalities in the coronary arteries, and polycystic structures could be seen at the position of mitral valve (Fig. 2), and more imaging evidence could not be provided due to poor image quality. Then we planned to perform valve tumor resection and mitral valve repair. However, no tumors and infectious vegetations were found on the valve leaflets during the operation. The cystic structure described by echocardiography was caused by prolapse due to leaflet degeneration (Fig. 3). In order to maintain the integrity of the leaflet structure, we performed folding repair for the mitral leaflet. The patient was recovered well after operation, and the result of echocardiography evaluation was satisfactory.

## Discussion

Echocardiography has developed rapidly since its appearance in the 1950s and is now the preferred choice for imaging occupying cardiac lesions. It provides high-quality, real-time images, which are invaluable in the evaluation of cardiac masses. Although transthoracic echocardiography is an excellent initial diagnostic technique for the evaluation and diagnosis of cardiac masses, transesophageal echocardiography provides superior image resolution and better visualization of cardiac masses in patients with poorly studied transthoracic echocardiography [2]. Some diseases with quite similar echocardiographic presentation need to differential diagnosis carefully, especially for cases where the symptomatic presentation cannot be clearly identified [3]. The treatment options varied widely between the two groups of potentially confusing diseases, such as the primary valve tumors and valve degeneration in this case.

Primary cardiac tumors are fairly uncommon, with an average incidence rate about 0.02%, while primary valvular tumors account for only 10% among them. The types that need differential diagnosis include myxoma, papillary elastic fibroma, lipoma, blood cysts, etc. [4–6]. The features of echocardiogram are often pedunculate, often a solitary mass, usually at the mid-portion of valve leaflets, and with a frond-like characteristic surface [7]. The primary valvular tumors need to be surgically removed if it is active, even for asymptomatic patients, because of the potential cerebral and cardiac embolization [8, 9]. Valvular degenerative disease due to the absence of fibrin in the valve leads to lengthy chordae tendineae and leaflet prolapse, which often causes different degrees of valvular regurgitation [10, 11]. In this case, the apex systolic murmur was consistent with the degree of echocardiographic mitral valve regurgitation.

Generally, only patients with severe mitral regurgitation combined with symptoms of cardiac insufficiency require surgery. This patient had severe mitral regurgitation before operation and the wrong judgment before operation did not cause any adverse effect. However, for suspected valve masses without valve regurgitation, more accurate evaluation and identification of primary valvular tumors and valve degenerative lesions is clearly necessary. For individual patient, it may require a combination clinical symptom, widen use of echocardiography and more auxiliary examinations such as three-dimensional echocardiography or a contrast study to make the optimal clinical decision.

## Supplementary Information

**Additional file 1.** Transesophageal echocardiography video-1.

**Additional file 2.** Transesophageal echocardiography video-2.

**Additional file 3.** Transesophageal echocardiography video-3.

## Data Availability

Not applicable.

## Abbreviations

CT: Computed tomography
MRI: Magnetic resonance imaging
CTA: Computed tomography angiography
